# Ferromagnetic Order at Room Temperature in Monolayer WSe_2_ Semiconductor via Vanadium Dopant

**DOI:** 10.1002/advs.201903076

**Published:** 2020-03-11

**Authors:** Seok Joon Yun, Dinh Loc Duong, Doan Manh Ha, Kirandeep Singh, Thanh Luan Phan, Wooseon Choi, Young‐Min Kim, Young Hee Lee

**Affiliations:** ^1^ Center for Integrated Nanostructure Physics (CINAP) Institute for Basic Science (IBS) Suwon 16419 Republic of Korea; ^2^ Department of Energy Science Sungkyunkwan University Suwon 16419 Republic of Korea

**Keywords:** gate‐controlled spintronics, gate tunable magnetism, magnetic domains, magnetic semiconductors, room temperature ferromagnetism, vanadium‐doped tungsten diselenide

## Abstract

Diluted magnetic semiconductors including Mn‐doped GaAs are attractive for gate‐controlled spintronics but Curie transition at room temperature with long‐range ferromagnetic order is still debatable to date. Here, the room‐temperature ferromagnetic domains with long‐range order in semiconducting V‐doped WSe_2_ monolayer synthesized by chemical vapor deposition are reported. Ferromagnetic order is manifested using magnetic force microscopy up to 360 K, while retaining high on/off current ratio of ≈10^5^ at 0.1% V‐doping concentration. The V‐substitution to W sites keeps a V–V separation distance of 5 nm without V–V aggregation, scrutinized by high‐resolution scanning transmission electron microscopy. More importantly, the ferromagnetic order is clearly modulated by applying a back‐gate bias. The findings open new opportunities for using 2D transition metal dichalcogenides for future spintronics.

## Graphical abstract

Magnetic domains are observed up to room‐temperature in semiconducting V‐doped WSe_2_ monolayer using magnetic force microscopy (MFM). Polarization of the magnetic tip and temperature‐dependent MFM measurements clarify the magnetic response of V‐doped WSe_2_. Vanadium is well substituted in the tungsten site without any aggregation in WSe_2_ structure. Importantly, the field tunability of the magnetic domains is demonstrated by performing MFM with applying back‐gate biases.

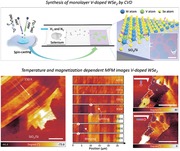

The magnetism of semiconductors can be tuned by modulating carrier concentration with a gate bias. While intrinsic magnetic semiconductors rarely exist in nature, the incorporation of magnetic dopants into innumerable semiconductors, called diluted magnetic semiconductors (DMSs), allows us to construct an inherent ferromagnetic (FM) state with spin‐polarized carriers at the Fermi level.^[^ ^1^, ^2^, ^3^, ^4^, ^5^, ^6^, ^7^, ^8^ ^]^ The most typical example is Mn‐doped GaAs, which exhibit a gate‐controlled magnetic hysteresis, yielding a large number of spintronic devices such as spin‐injection sources and memory devices.^[^ ^2^, ^9^, ^10^, ^11^, ^12^, ^13^ ^]^ Nevertheless, the Curie temperature (*T*_c_) of ferromagnetic transition in magnetic semiconductors is scarcely accessible to room‐temperature (RT), precluding the use of these materials to practical implementations.^[^ ^2^, ^3^, ^4^, ^5^, ^14^ ^]^ The ferromagnetic state in magnetic metal‐doped oxides and nitrides is available at RT but is localized to aggregated metal oxide/nitride nanoparticles without a long‐range magnetic order.^[^ ^4^ ^]^

The ferromagnetic state in van der Waals 2D materials has been observed recently in the monolayer limit.^[^ ^15^, ^16^, ^17^, ^18^, ^19^, ^20^, ^21^ ^]^ Intrinsic CrI_3_ and CrGeTe_3_ semiconductors reveal ferromagnetism but the *T*_c_ is still low below 60 K.^[^ ^20^, ^21^ ^]^ In contrast, monolayer VSe_2_ and MnSe_2_ are ferromagnetic metals with *T*_c_ above RT but incapable of controlling its carrier density.^[^ ^22^, ^23^ ^]^ Moreover, the long‐range ferromagnetic order in doped diluted chalcogenide semiconductors has not been demonstrated at RT.^[^ ^24^, ^25^, ^26^, ^27^, ^28^ ^]^ The key research target is to realize the long‐range order ferromagnetism, *T*_c_ over RT, and semiconductor with gate tunability. Here, we unambiguously observe tunable magnetic domains by a gate bias above RT in diluted V‐doped WSe_2_, while maintaining the semiconducting characteristic of WSe_2_ with a high on/off current ratio of five orders of magnitude.

**Figure** **1**a illustrates the schematic for the synthesis of V‐doped monolayer WSe_2_ via chemical vapor deposition (CVD). A metal precursor solution prepared by mixing V and W liquid sources at a given atomic ratio was spin‐coated on SiO_2_ substrate and the substrate was introduced into the CVD chamber with selenium. The metal precursors get decomposed into metal oxides at growth temperature, resulting in monolayer V*_x_*W_1−_ *_x_*Se_2_, followed by selenization. The atomic ratio of V to W sources in precursor solution can be precisely controlled from 0.1% to 40%, while the hexagonal flakes are retained in a monolayer form (the optical image in Figure 1a; Figure S1, Supporting Information). Meanwhile, the dendritic and multilayer flakes are partially generated at higher V‐concentration. The V atoms are incorporated into monolayer WSe_2_ with V/W contents similar to nominal values, as confirmed by X‐ray photoelectron spectroscopy (Figure S2, Supporting Information). With low V‐doping concentration, the hexagonal V‐doped WSe_2_ flake is a single crystal confirmed by previous TEM study.^[^ ^29^ ^]^

**Figure 1:**
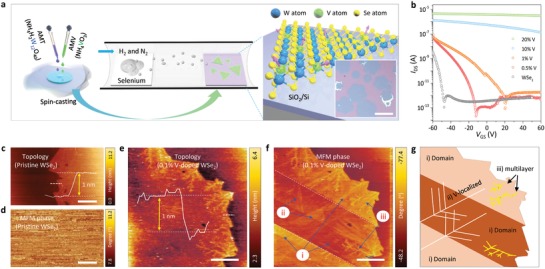
Synthesis of semiconducting V‐doped monolayer WSe_2_ and ferromagnetic characteristics. a) Schematic of synthesis of V‐doped WSe_2_ by mixing liquid W with V precursors. The inset shows optical image of CVD‐grown V‐doped WSe_2_ monolayer. Scale bar, 50 µm. b) Source‐drain current (biased at 1 V) with the gate bias for V‐doped WSe_2_ field‐effect transistors with various V‐doping concentrations. c) Topography and d) MFM phase images of pristine WSe_2_ at RT. Scale bar, 10 µm. e) Topography and f) MFM phase images of 0.1% V‐doped WSe_2_ at RT. Scale bar, 10 µm. g) The schematic typical features observed in MFM phase images.

To study the doping effect of vanadium to the electronic properties of WSe_2_, field effect transistors (FETs) of V‐doped monolayer WSe_2_ were fabricated (Figure 1b). The CVD‐grown pristine WSe_2_ manifests a p‐type semiconductor with a threshold voltage at −50 V. The threshold voltage is shifted to −10 V for 0.5% V‐doped sample and further increased to +20 V for 1% V‐doped sample, while retaining high on/off ratio of ≈10^5^ and distinct p‐type behavior. In contrast, the on/off ratio is significantly reduced for >10% doping samples due to the formation of heavily degenerate V‐doped WSe_2_.

Since V‐doped WSe_2_ monolayer sample has an extremely small mass (10^−6^ g cm^−2^), mass‐dependent magnetic characterization methods such as vibrating sample magnetometer (VSM) and superconducting quantum interference device (SQUID) are not adequate due to the detection limit. The signal from V‐doped WSe_2_ monolayer sample could be below the detection limit of these systems (Figure S3, Supporting Information). Furthermore, such small signals, if detectable, may be obscured by the artifacts of magnetic impurities (e.g., particles with iron content, kapton tape for holding samples) during sample preparation and measurement processes.^[^ ^30^ ^]^

Meanwhile, the magnetic force microscopy (MFM) is surface‐sensitive and can detect very small magnetic dipole moments with a nanometer scale (Figure S3, Supporting Information). Therefore, we investigate the existence of magnetic order of V‐doped WSe_2_ monolayer by using the MFM. Figure 1c–f are the tapping‐mode topography and MFM phase images with a Co–Cr tip of pristine WSe_2_ (Figure 1c,d) and 0.1% V‐doped (Figure 1e,f) WSe_2_ monolayer at 300 K. No distinctive feature was observed in MFM phase, indicating no ferromagnetic order in pristine WSe_2_ (Figure 1d). In contrast, three distinct features (marked as numbers) are eminent in MFM phase image at 0.1% V‐doped WSe_2_ (Figure 1f and the schematic in Figure 1g): i) large domains with phase contrast separated by domain walls (white dotted line), ii) the dendritic patterns in monolayer flake, and iii) multilayer dendrites, correlated with the topography image in Figure 1e.

Ferromagnetic domain stripes of the MFM phase are more clearly manifested at 150 K (**Figure** **2**a). The domain stripes merge (region a) and split (region b) as temperature increases, strongly implying the domain features originated from magnetic response (Figure 2b; Figure S4, Supporting Information). The distinct magnetic phase becomes ambiguous above RT (Figure 2c) but the magnetic domain walls still retain remanent up to 420 K. To elucidate how the magnetic domains are modulated with V‐doping concentration, we further conducted MFM for 0.5% and 2% V‐doped WSe_2_ (Figure 2d; Figure S5, Supporting Information). A strong contrast phase within the flake still emerges from the 0.5% sample up to 420 K with dendritic patterns. Similar dendritic patterns were observed in the photoluminescence (PL) mapping (Figure 2e). The PL intensity quenching and peak red‐shift in region (2) and (3) with respect to region (1) originate from the formation of positive trions and charge screening (Figure 2f). This is ascribed to inhomogeneous V‐doping concentration in different regions. This does not necessarily imply strong correlation of the magnetic domains to the PL patterns (electrostatic contribution). The PL measurements are also consistent to Raman mapping (Figures S6 and S7, Supporting Information).

**Figure 2:**
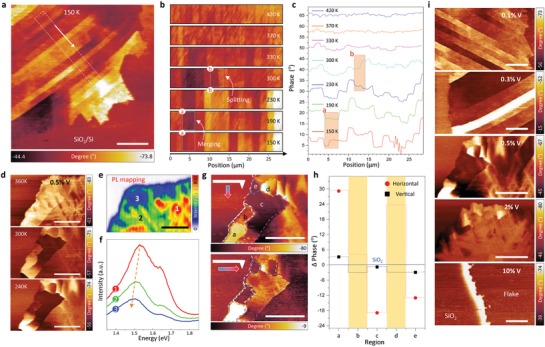
MFM with magnetic domains in V‐doped WSe_2_. a) MFM phase image of 0.1% V‐doped WSe_2_ taken at 150 K. b) Temperature‐dependent transition of magnetic domains and c) related phase profiles in the white‐dotted box in (a). d) Temperature‐dependent MFM phase images of 0.5% V‐doped WSe_2_. e) Photoluminescence mapping and f) the corresponding spectra at different positions numbered in (e). g) MFM response of 0.5% V‐doped WSe_2_ with different magnetized directions of tip at 240 K. h) Average phase signal of regions indicated by the letters in (g). The phase value for SiO_2_ is set to zero for the reference. i) Magnetic domains of V‐doped WSe_2_ with different V‐concentrations. All scale bars, 10 µm.

It is worth noting that MFM signal using magnetic tip has been detected in the exfoliated MoS_2_ and graphene flakes.^[^ ^31^ ^]^ However, the electrostatic force was the main contribution in those MFM measurements.^[^ ^32^ ^]^ To exclude the electrostatic contribution and further confirm the magnetic nature of the observed domains, we carried out the MFM measurements with the tip magnetized vertically and horizontally. Although the magnetic polarization of horizontally magnetized tip is quite complicated due to tip morphology, the horizontal and vertical MFM phases can be quantitatively distinguished. The amplitude of the magnetic signals is much stronger with vertical magnetized tip than with horizontal one (Figure 2g,h). Additionally, the sign of the phase (compared to the SiO_2_ background) is inverted in some domains. For example, the contrast of region (b) is positive with vertical magnetized tip, whereas it is negative with horizontal one. This indicates the magnetic force changes from repulsive to attractive force, which is also solid evidence for the magnetic response. Tip‐dependent MFM measurement was further performed to ensure magnetism of the V‐doped WSe_2_ (Figure S8, Supporting Information).

Figure 2i summarizes the magnetic response of V‐doped WSe_2_ samples with different V‐doping concentrations. At low doping concentration (0.1% and 0.3%), the magnetic domains were retained with a line stripe. We notice that the stripe‐like domains are observed in different crystal and polycrystalline structures.^[^ ^33^, ^34^ ^]^ In addition, the magnetic domains were transformed to a polygon shape at high V‐doping concentration (0.5% and 2%). Therefore, we expect no distinct correlation between the magnetic domain and crystal structures. We note that that the area of the magnetic domain becomes smaller as the V‐doping concentration increases and the phase contrast was eventually not appreciable at 10%. The magnetic domains are also sensitive under ambient conditions (Figure S9, Supporting Information). The monolayer sample is directly exposed to environment and its electronic states can be dramatically influenced. Such a variation of electronic structures or any charge transfer can give rise to the change of magnetic properties.^[^ ^35^ ^]^ The passivation of V‐doped WSe_2_ samples by Al_2_O_3_ allows us to minimize the change of inherent magnetic domains (Figure S10, Supporting Information).

To estimate *T*_c_ from MFM measurements, we extracted the root‐mean‐square phase deviation from MFM images (Figure S5c, Supporting Information).^[^ ^36^ ^]^ *T*_c_ can be determined by the slope change of the curves, that is, ≈360, ≈270, and ≈220 K for corresponding 0.1%, 0.5% and 2% V‐doped samples. Complicated *M*–*T* curve are observed, indicating the presence of multiphase transition including spin‐glass rather than simple monotonic ferromagnetic ordering. While magnetic phases vary with different positions of the samples with large error bars, we can still see clearly qualitatively the Curie temperature dependence with V‐doping concentration.

To investigate the atomic structure of V‐doped WSe_2_, we conducted the annular dark field scanning transmission electron microscopy (ADF‐STEM) for 0.1% and 2% V‐doped WSe_2_ (**Figure** **3**). A well‐crystallized 2H–WSe_2_ structure is clearly demonstrated with W (bright) and Se (gray) sites with additional V (dark) atoms, as is consistent with the intensity profile with simulated images (Figure 3a–d). Well‐distributed V atoms in both samples without any clustering indicates that the magnetic properties of V‐doped WSe_2_ did not originate from phase segregation of vanadium but rather result from interaction between V atoms via host WSe_2_. Up to V‐10% doping concentration, V atoms are well distributed without being clustered in the host WSe_2_ (Figure S11, Supporting Information). We analyzed the concentration for 0.1% and 2% V‐doped samples (Figure 3e,f). Three impurities are clearly identified after Wiener‐filter false coloring (Figure S12, Supporting Information): Se‐vacancy in WSe_2_ (WSe), V‐substitution into W site in WSe (VSe), and VSe_2_. Five STEM images of each sample were thoughtfully analyzed for reliable statistics (Figure S13, Supporting Information). The real V‐concentration of nominal 0.1% and 2% V‐doped samples are similar to 0.1 ± 0.01% and 1.0 ± 0.07%, respectively (Figure S14, Supporting Information).

**Figure 3:**
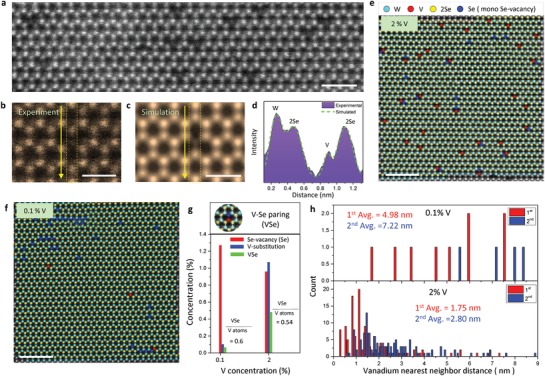
Atomic structure of V‐substituted WSe_2_ observed by STEM. a) ADF‐STEM images of V‐doped monolayer WSe_2_. Scale bar, 1 nm. b) Experimental, c) simulated images, and d) their intensity profiles for V‐doped WSe_2_. Scale bar, 5 Å. False‐color Wiener‐filtered STEM images of e) 2% and f) 0.1% V‐doped WSe_2_. Scale bar, 2 nm. Statistical analysis of V‐doped WSe_2_ for g) V substitution, Se vacancies, VSe species and h) vanadium nearest neighbor distances (h).

Chalcogen defects can influence magnetic properties of TMDs.^[^ ^37^ ^]^ However, Se‐vacancy concentration is irrespective of the V‐composition (approximate 1% or 10^13^ cm^−2^ in both 0.1% and 2% samples). This strongly implies that the RT‐ferromagnetism is not attributed by Se‐vacancies. The role of V‐substituted forms (VSe or VSe_2_) is as yet unclear for the magnetism of V‐doped WSe_2_. However, the ratios of VSe to total V atoms (VSe + VSe_2_) are similar in both 0.1% and 2% V‐doped samples (Figure S15, Supporting Information), indicating that VSe or VSe_2_ doping concentration is closely correlated to each other for discernible *T*_c_. We next explore the first and second nearest neighbor distances between V atoms (Figure 3h). The average V–V neighbor distance is much longer in 0.1% (50 Å, or ≈15 unit cells) than in 2% (18 Å, or ≈5 unit cells) V‐doped samples.

Gate‐tunable magnetic property is crucial evidence to demonstrate the ferromagnetic semiconductor. We performed the MFM measurements under applying gate biases (**Figure** **4**a). The phase contrasts by ferromagnetic domains of 0.1% V‐doped WSe_2_ with different gate biases from −10 to 20 V are shown in Figure 4b. This demonstrates an apparent variation of the contrast between domains with gate biases (Figure S16, Supporting Information). The deviation of contrast between domains is almost negligible at a negative gate bias of −10 V. As the gate bias is shifted toward the positive gate bias to 15 V, the phase contrast becomes distinct (Figure 4c). Interestingly, the phase deviation drops at a high gate bias of >15 V. The non‐monotonic change of the phase deviation through the gate bias again excludes the electrostatic artifact in our gate‐dependent MFM measurement (if the phase deviation is solely contributed by electrostatic force, it should be monotonic with the gate bias). Furthermore, the faint magnetic domains at the negative bias (−10 V) at room temperature emerged predominantly at 150 K (Figure 4d,e). This strongly indicates the gate‐tunable magnetic properties in V‐doped WSe_2_ samples.

**Figure 4:**
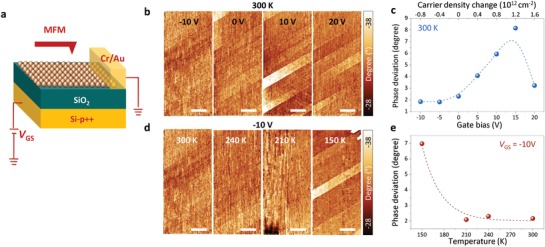
Gate‐tunable magnetic properties and band structure of V‐doped WSe_2_. a) Schematic of experimental arrangement for gate‐dependent MFM measurements. b) Gate‐dependent MFM images and c) their phase deviation for 0.1% V‐doped WSe_2_. d) Temperature‐dependent MFM images and e) their phase deviation at −10 V of gate bias. All scale bars, 10 µm.

In summary, we have successfully synthesized V‐doped WSe_2_ in monolayer, which reveals RT dilute ferromagnetic semiconductors. The existence of the ferromagnetic order is confirmed at microscopic scale by MFM. Furthermore, the magnetic order can be modulated with back‐gate bias, which implies the possibility of the Ruderman–Kittel–Kasuya–Yoshida interaction (or Zener model) by establishing the long‐range ferromagnetic order in V‐doped WSe_2_ monolayer through free hole carriers. Our work opens a direct route to demonstrate practical applications of TMDs in spintronic devices at RT.

## Experimental Section

Experimental methods are presented in detail in Supporting Information

## Conflict of Interest

The authors declare no conflict of interest.

## Supporting information

Supporting Information
