# Supplementary material for: Ferromagnetic Order at Room Temperature in Monolayer WSe2 Semiconductor via Vanadium Dopant
Source: Adv Sci (Weinh). 2020 Mar 11;7(9):1903076. doi: 10.1002/advs.201903076 (PMC7201245; doi:10.1002/advs.201903076)
Supplement: Supplementary file 1 — Supporting Information [file ADVS-7-1903076-s001.pdf]

Copyright WILEY-VCH Verlag GmbH & Co. KGaA, 69469 Weinheim, Germany, 2020.

Supporting Information

for *Adv. Sci.,* DOI: 10.1002/advs.201903076

Ferromagnetic Order at Room Temperature in Monolayer WSe_2 Semiconductor via Vanadium Dopant

Seok Joon Yun, Dinh Loc Duong,* Doan Manh Ha, Kirandeep Singh, Thanh Luan Phan, Wooseon Choi, Young-Min Kim, and Young Hee Lee**

Copyright WILEY-VCH Verlag GmbH & Co. KGaA, 69469 Weinheim, Germany, 2020.

Supporting Information

Ferromagnetic order at room temperature in monolayer WSe_2 semiconductor via vanadium dopant

Seok Joon Yun[†*], *Dinh Loc Duong*[†*], *Doan Manh Ha, Kirandeep Singh, Thanh Luan Phan, Wooseon Choi, Young-Min Kim, and Young Hee Lee*[*]

[†]These authors contributed equally to this work.

S1. Synthesis of V-doped WSe_2 monolayer by chemical vapor deposition

Metal precursor solutions were prepared by mixing four types of water-based solution defined as A, B, C and D. A: tungsten precursor, where ammonium metatungstate hydrate (AMT) [$(NH_4)_6H_2W_{12}O_{40}{\bullet}xH_2O$: Sigma-Aldrich, 463922] was dissolved in deionized (DI) water (0.1 gram of AMT in 5 ml of DI water), B: vanadium precursor, where ammonium metavanadate (AMV) [NH_4VO_3: Sigma-Aldrich, 573884] was dissolved in DI water (0.3 gram of AMV in 50 ml of DI water), C: promoter for monolayer, where sodium hydroxide (Sigma-Aldrich, 306576) as a promoter for increasing the monolayer portion was dissolved in DI water (0.1 gram of NaOH in 30 ml of DI water). D: medium solution, where OptiPrep density gradient medium (Sigma-Aldrich, D1556, 60% (w/v) solution of iodixanol in water) was introduced as a medium to mix the promoter and precursor for a better spin casting process. Such solutions were mixed in given ratios to control doping degrees and then the mixed solution was coated onto an SiO_2/Si wafer by a spin-casting process at 3000 rpm for 1 min. During CVD process, AMT and AMV were decomposed into respective WO_x and VO_x, which were reacted to selenium with the presence of hydrogen gas at 750 °C to form monolayer $W_{1-x}V_xSe_2$. The presence of hydrogen gas is important due to the low reactivity of metal oxides with selenium.

S2. Magnetic force microscopy for magnetic domains

The magnetic domains were measured by using the E-Sweep atomic force microscopy (Hitachi) under the lift mode. Samples were *in situ* annealed at 300 °C in a vacuum of 5.10^{-6} Torr for 2 hours prior to measurements. All the measurements were always performed at the same vacuum level. The Co-Cr coated NSC36 tips (MikroMasch) were used with a controlled Q-factor approximately 2,000. The magnetization of the tip was controlled by using a permanent magnet (SI Figure S18).

S3. Origin of MFM signals and phase deviation calculation from MFM image

The response of the MFM phase contrast originates from the long-range interaction between tips and samples. The signal is in general strong when samples and tips are both ferromagnetic. Furthermore, it has been proved that the MFM phase contrast is also strong when tips are paramagnetic and samples are ferromagnetic[1]. In our case, ferromagnetic tips were used. Therefore, a strong signal of MFM phases indicates either ferromagnetic or paramagnetic states in V-doped WSe_2. For our analysis, the ferromagnetic domains have a well-defined shape with domain walls, which can give rise to both negative (attractive force) and positive (repulsive force) contrast compared to the phase (contrast) of the SiO_2 background as reported previously[2]. In contrast, there is no well-defined walls in the paramagnetic state and the magnetic response of paramagnetism is always negative (attractive force) compared to the SiO_2 background.

The phase deviation value of MFM image is defined as $\delta f_{\mathrm{dev.}} = \sqrt{\sum \frac{(f_i - f_0)^2}{(N-1)}}$ where f_i is phase value at each point, f_0 is the average of phase value, N is the total number of points in MFM images.

S4. Device fabrication and measurement

V-doped monolayer WSe_2 was transferred onto a highly *p*-doped silicon substrate with a 300-nm-thick oxide by the HF-based wet transfer method. The metal electrodes for probe contacts were patterned on the samples by an e-beam lithography followed by an e-beam deposition of Pd/Au (10/40 nm). All electrical measurements were performed under high vacuum (~10^{-6} Torr) using a Keithley 4200 SCS system.

S5. Transmission electron microscopy and specimen preparation

TEM and ADF-STEM were taken by a probe aberration-corrected JEM ARM 200F machine, operated at 80 keV for high-resolution TEM measurements. We set the imaging time within 10 seconds under a high-magnification STEM node to minimize the beam damage on monolayer samples. For transferring sample to the TEM grid, poly(methyl methacrylate) (PMMA C4, MicroChem) was coated onto samples as a supporting layer and then immersed into diluted hydrofluoric acid for detaching V-doped WSe_2 from the wafer by etching silicon oxide. The PMMA-supported samples were transferred to the TEM grids (PELCO, 200 mesh, copper, 1.2-μm holes). The PMMA was then removed by acetone. To avoid the polymerization during STEM imaging, the grids were annealed at 180 °C in high vacuum chamber (7.5×10^{-5} Torr) for 24 hours prior to the TEM analysis.

S6. Optical measurement

X-ray photoemission spectroscopy (K-Alpha, THERMO FISHER) was employed to characterize elemental composition of V-doped WSe_2. The confocal Raman and photoluminescence measurement were conducted by using a Nanobase system with an excitation wavelength of 532 nm under high vacuum (10^{-6} Torr).

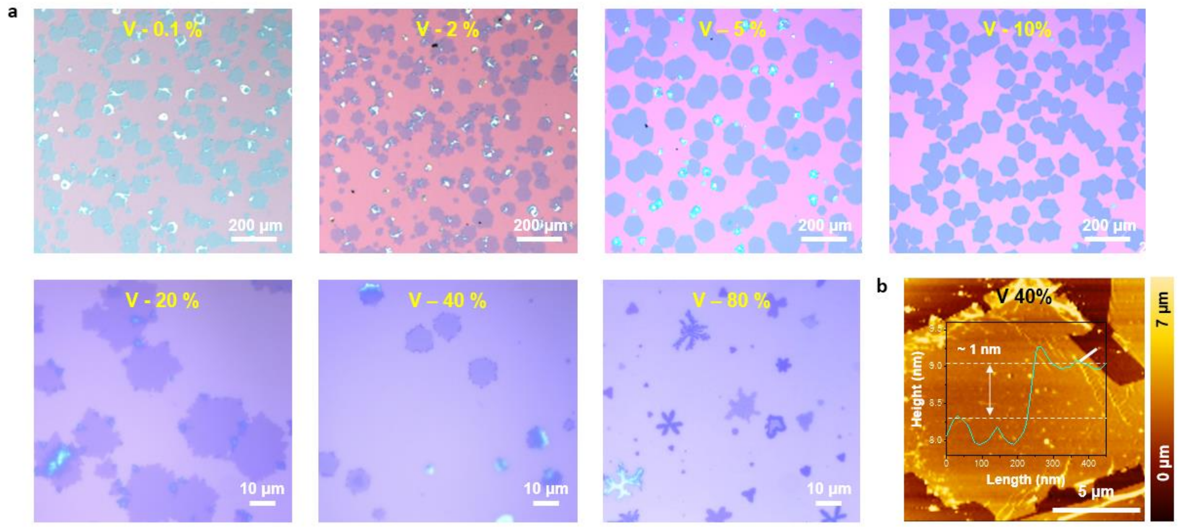

Figure S1. Shape variation of $V_xW_{1-x}Se_2$ flake with vanadium concentration. (a) Optical images of $V_xW_{1-x}Se_2$ alloys with different mixing atomic ratios between W and V (nominal value). Dendritic shape and thicker flakes were generated in the 80% V-doped sample. (b) AFM image and its height profile along the white line of the 40% V-doped WSe_2. A height of 1 nm indicates that monolayer $V_xW_{1-x}Se_2$ was grown up to 40% V-concentration.

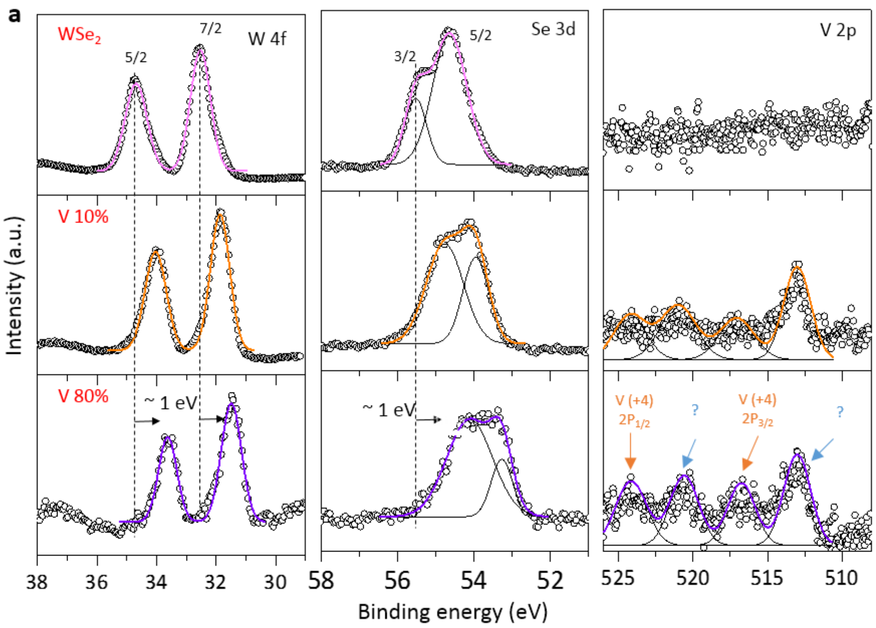

Figure S2. X-ray photoelectron spectroscopy measurement for V-doped WSe_2. X-ray photoelectron spectroscopy spectra of pure WSe_2, 20%, and 80% V-doped WSe_2. The real concentration of 20% and 80% V-doped samples are 17% and 67%, respectively. The red-shifts of W 4f and Se 3d indicate the *p*-doping effect of vanadium on WSe_2. Unknown peaks are seen in the V 2p spectra, which can be ascribed to V (+2) or 2H-VSe_2.

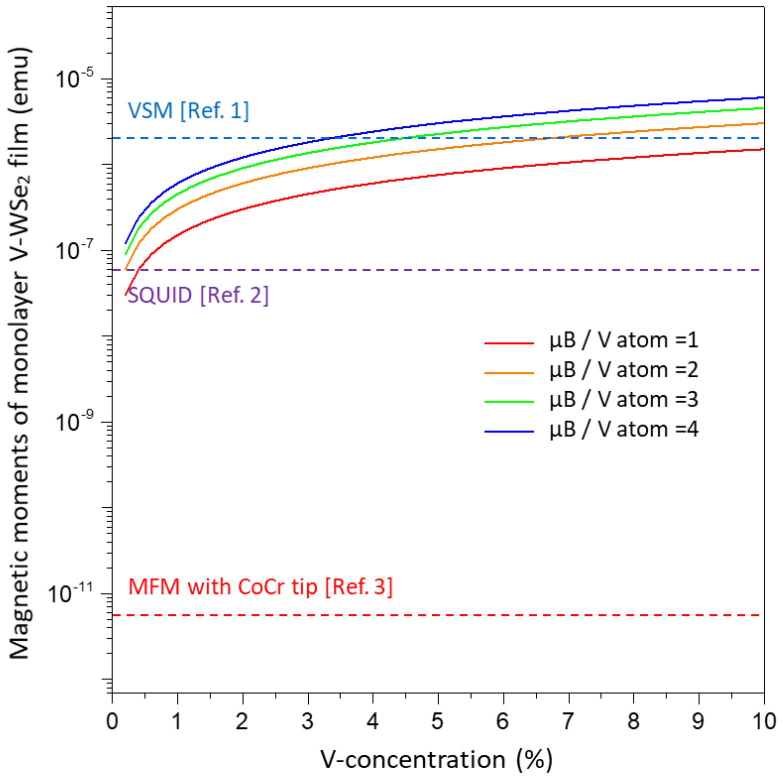

Figure S3. Estimated magnetic moments of monolayer V-doped WSe_2 film with different concentrations and Bohr magnetron values of V-dopant. The magnetic moments (M (emu)) are calculated on the assumption that magnetic moments are only attributed by V atoms, where M (emu) = Sample area (4 × 4 cm^2) × V-concentration (%) × density of metal sites in WSe_2 lattice (1.024 × 10^{12} cm^{-2}) × Bohr magnetron of V atom (μB) * converting parameter (emu $μB^{-1}$). Among the various magnetic characterization tools (VSM, SQUID, and MFM), the MFM can be most suitable to characterize magnetic properties of monolayer V-WSe_2 sample because of its high surface sensitivity and detection limit.

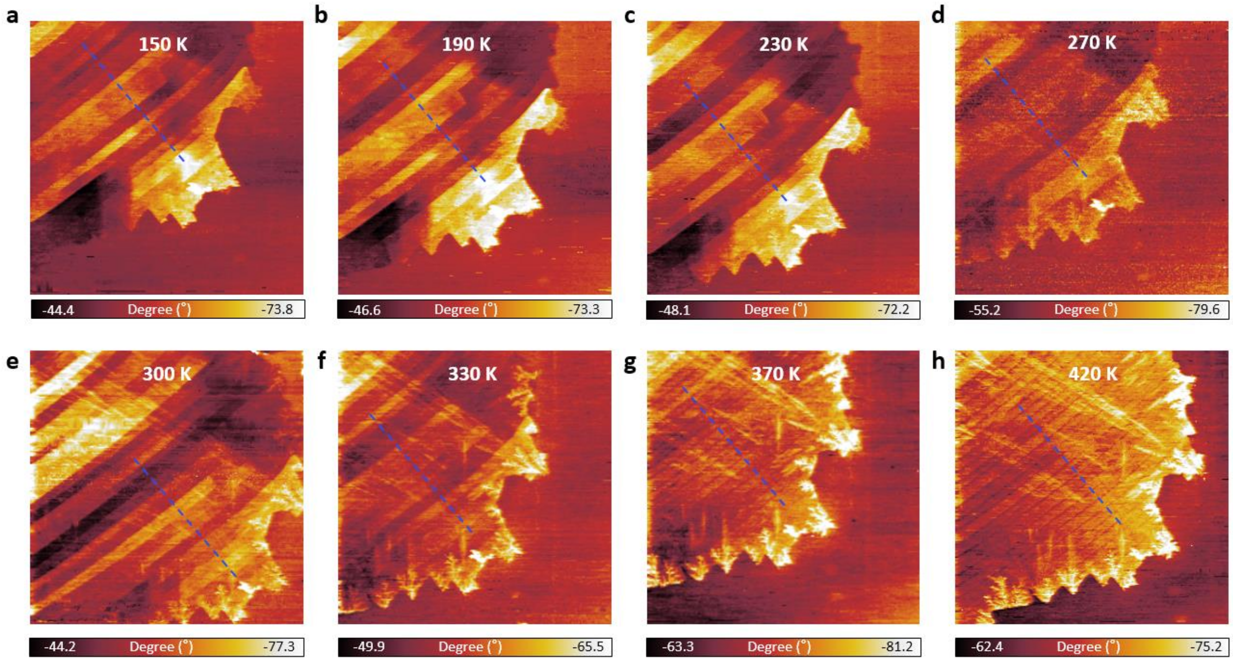

Figure S4. Evolution of MFM phase contrast with temperature in 0.1% V-doped WSe_2. (a-h) The domain strips merge and split with temperature. This temperature-dependent transformation of the MFM phase is a clear evidence for the magnetic response of V-doped WSe_2. Domain strips are merging and their contrasts become faded as temperature increases while dendritic patterns and multilayers regions appear with high phase signals.

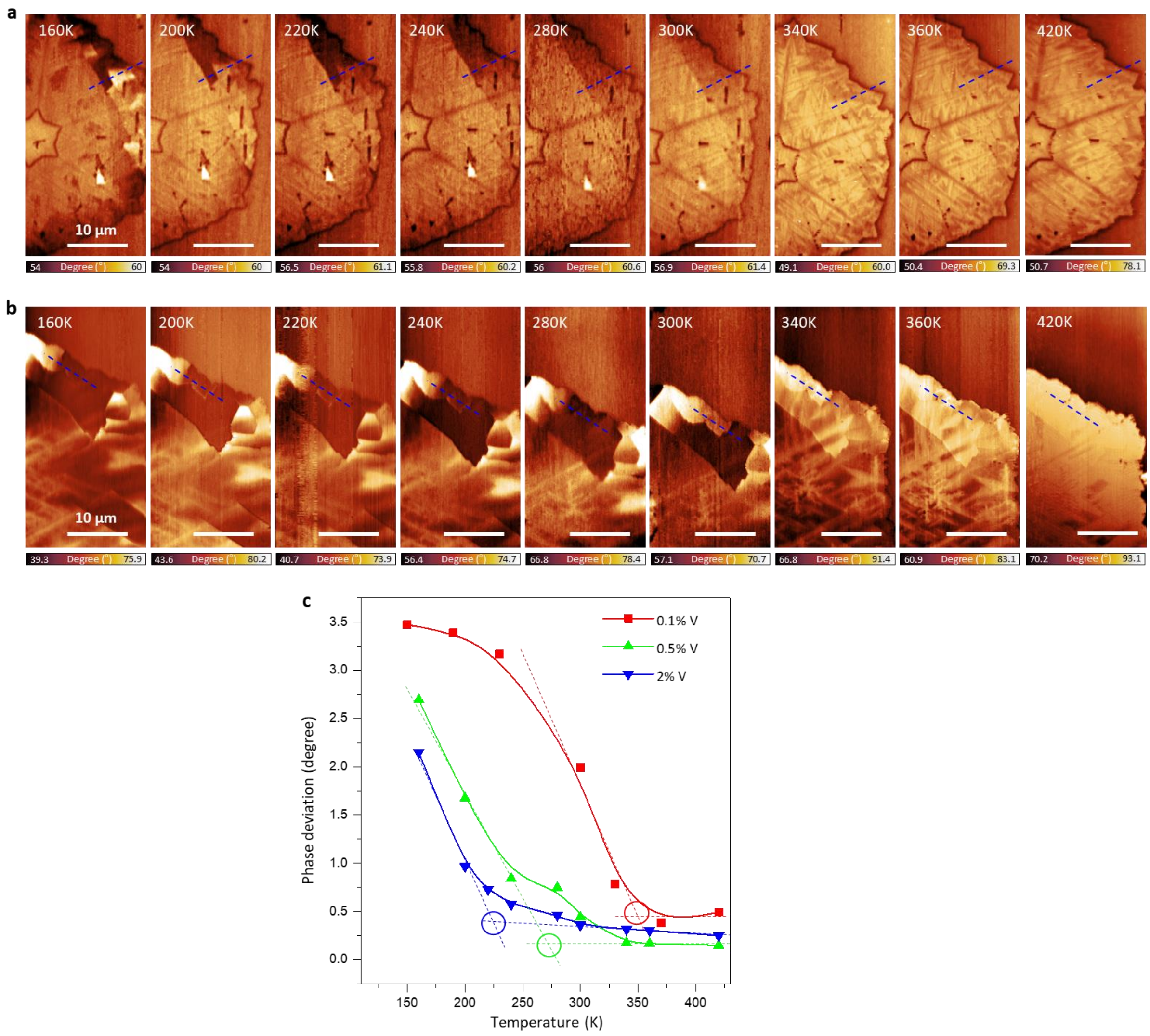

Figure S5. Evolution of MFM phase contrast with temperature in 2% and 0.5% V-doped WSe_2. Shown are the MFM phase images of (a) 2% and (b) 0.5% V-doped WSe_2. (c) Temperature dependence of the root-mean-square phase deviation from MFM phase of V-doped WSe_2 at different V-concentrations. Arbitrary magnetic domains with dashed lines are chosen for analysis in Figure S4 and S5.

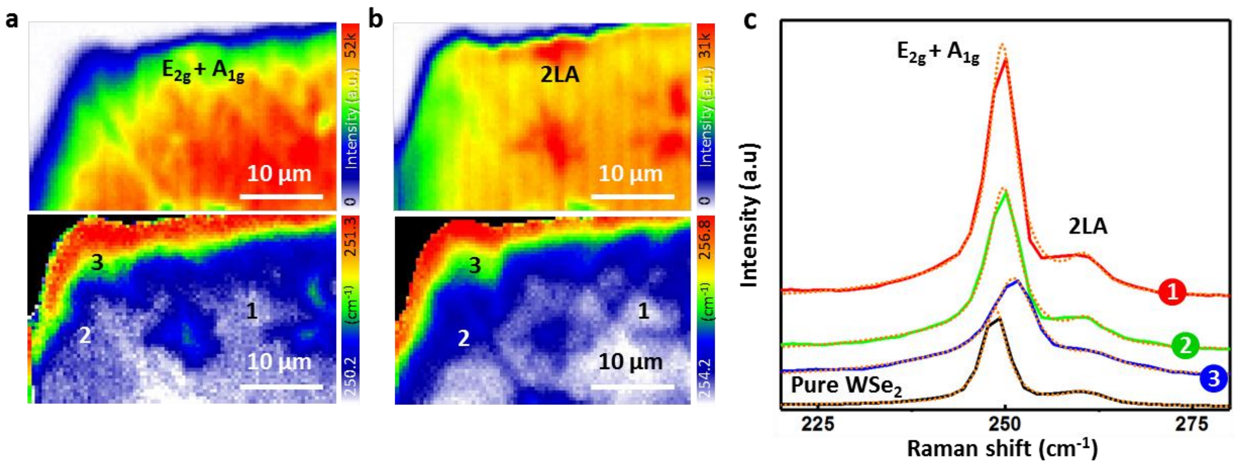

Figure S6. Raman mapping images of 0.5% V-doped WSe_2 measured at RT. (a) Raman intensity (top) and peak position (bottom) mapping of the E_{2g} + A_{1g} mode. (b) Raman intensity (top) and peak position (bottom) mapping of the 2LA mode. (c) Raman spectra from numbered regions in (a and b). The Raman intensity and position mapping images reveal non-uniformity of V-distribution in V-doped WSe_2 within the flake. The blue-shifted E^1_{2g} + A_{1g} mode could be attributed by V-incorporation.

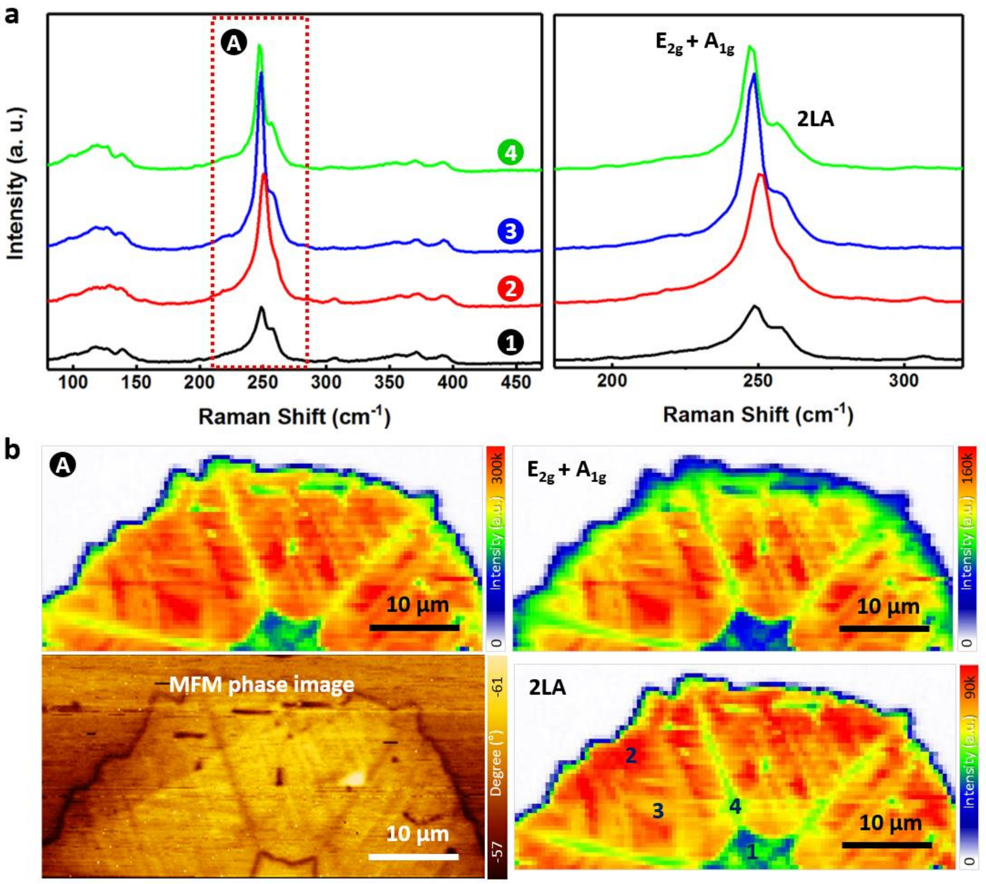

Figure S7. Comparison of the MFM phase image with Raman mapping of 2% V-doped WSe_2. (a) Raman spectra from numbered regions in (b). (b) Raman intensity mapping of E^1_{2g} + A_{1g} (top right), 2LA (bottom right) and the total intensity of both peaks (top left). Raman mapping images for 2% V-doped sample again imply a non-uniformity of V atoms in flakes.

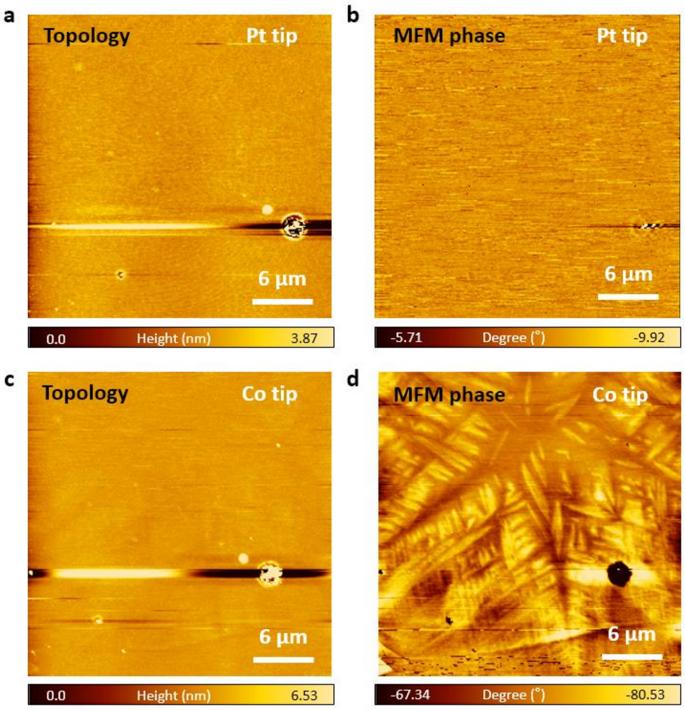

Figure S8. Negligible contribution of electrostatic force in the MFM images. The topology and MFM images of the 2% V-doped WSe_2 using (a, b) Pt and (c, d) Co-Cr tips. Since electrostatic and magnetic force can be detected in MFM measurements, we conducted MFM for V-doped WSe_2 with non-magnetic Pt tip and a ferromagnetic Co-Cr tip to discern the contribution of electrostatic force in the MFM image. No discernable contrast in the MFM phase image of V-doped WSe_2 with the Pt tip implies a negligible contribution of the electrostatic force in MFM phase images.

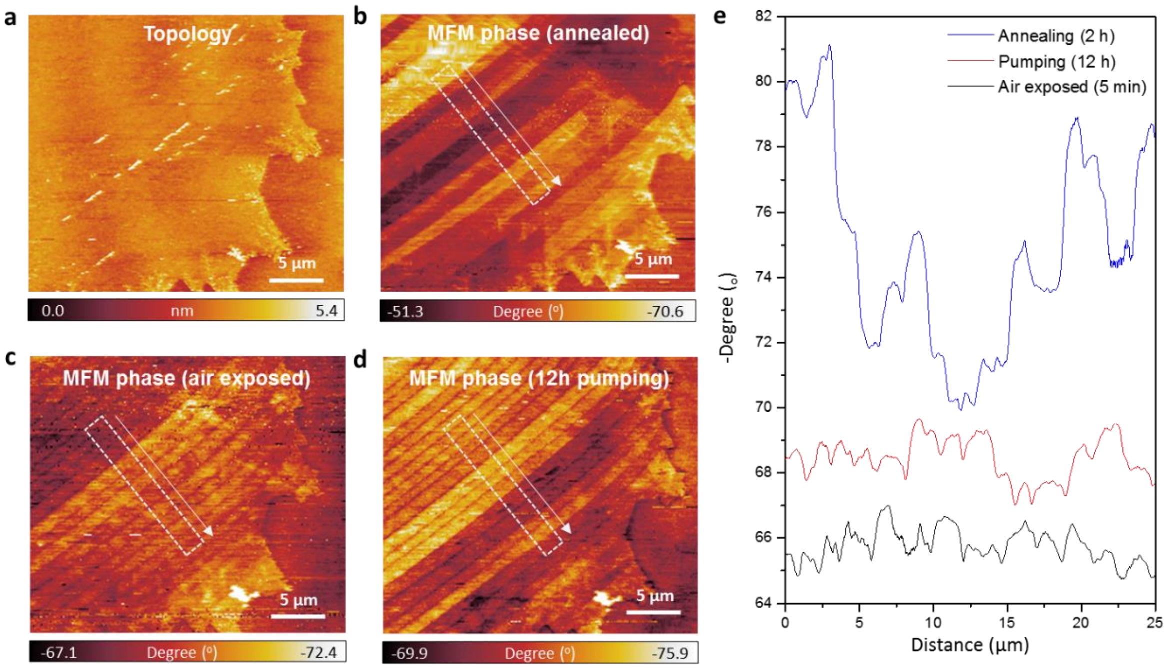

Figure S9. Effect of ambient conditions on the MFM measurement. (a) Topology and (b) MFM phase images of 0.1% V-doped WSe_2 after annealed at 300 °C for 2 hours, (c) exposing the sample in air for 5 minutes, (d) 12 hours pumping at 10^{-6} Torr. (e) Phase profiles extracted from the white dashed boxes in (b-d). The MFM phase contrast is disrupted when the sample is exposed to air.

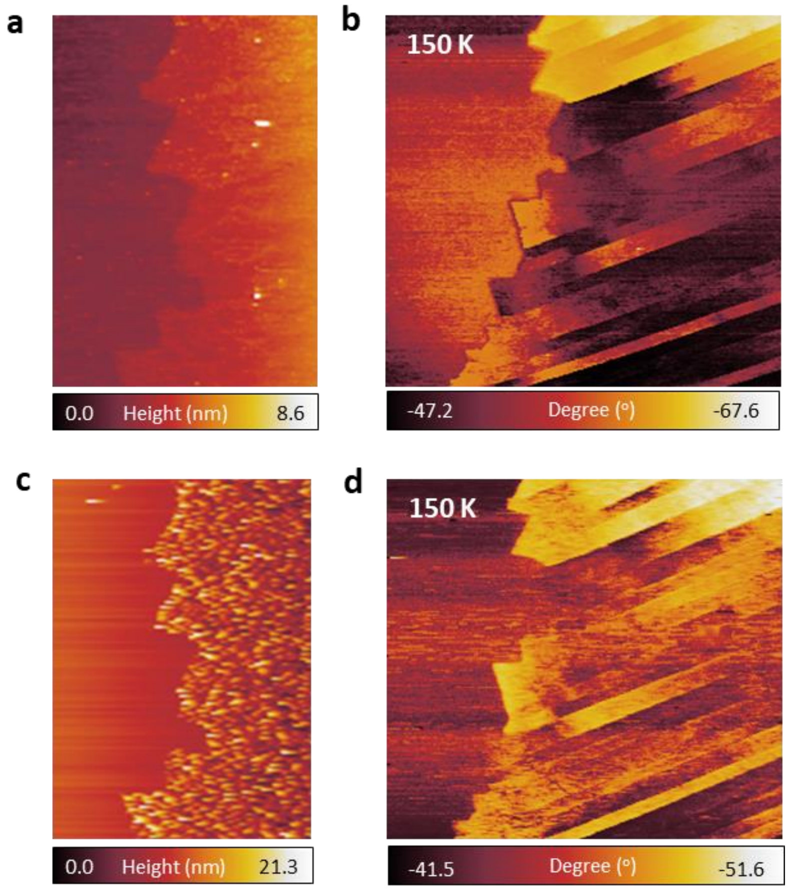

Figure S10. Effects of Al_2O_3 passivation on MFM measurement. (a) Topology and (b) MFM image of 0.1% V-doped WSe_2 measured at 150K and 10^{-6} Torr. The sample was passivated by a 15 nm Al_2O_3 layer and measured again without annealing process. Although the Al_2O_3 is not uniformly coated as shown in (c) topology image, similar magnetic domains were observed in (d) MFM image, indicating that the passivation can isolate the samples from the ambient conditions.

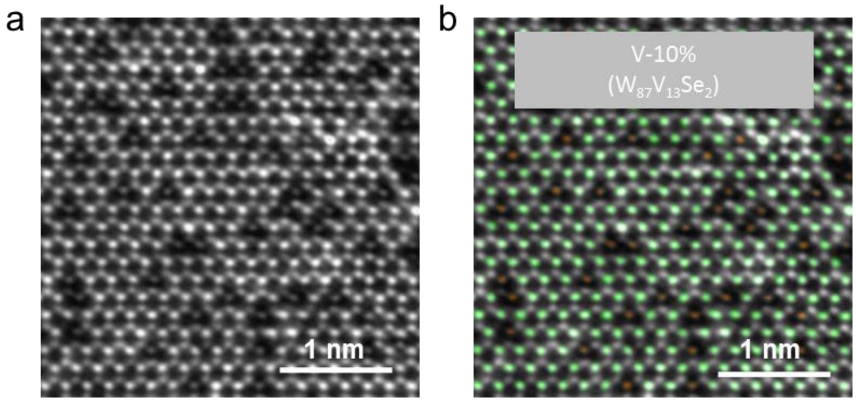

Figure S11. (a-b) STEM images of V-10% doped WSe_2 after (a) Gaussian-blur filtering and (b) false coloring. W (green), V (brown), and Se (white) atoms are clearly distinguished after the false coloring process.

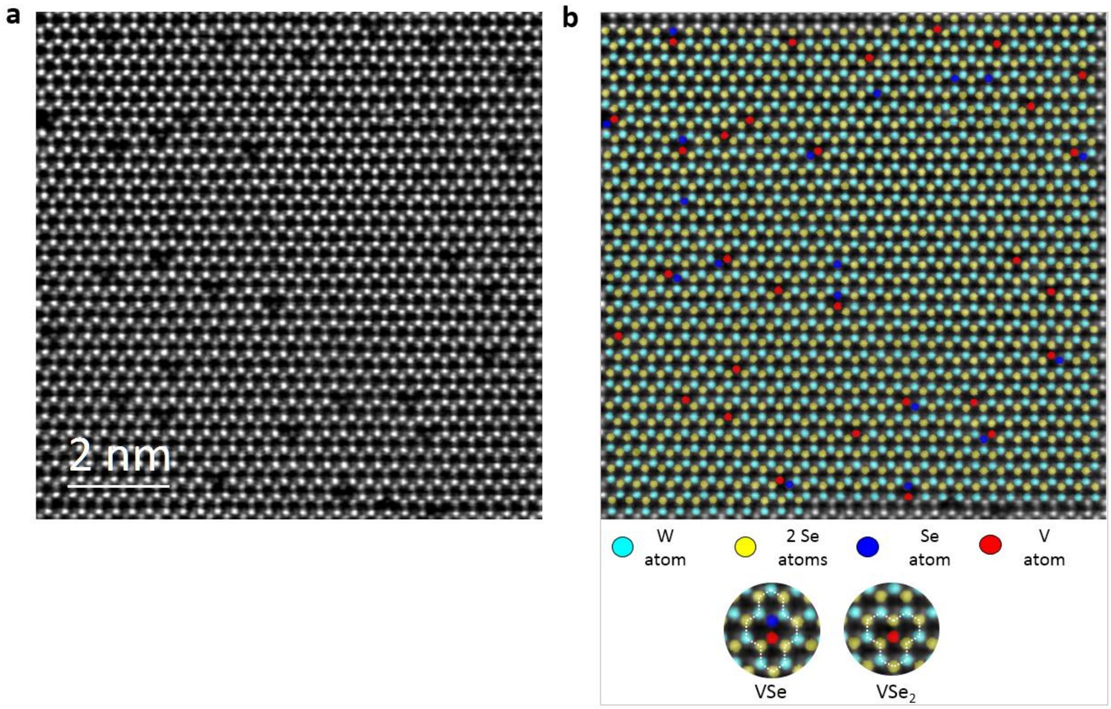

Figure S12. (a) Weiner-filtered and (b) false-colored ADF-STEM images of 2% V-doped monolayer WSe_2. The visibility and contrast between atoms were enhanced by applying the 'Local 2D Wiener/Difference Filter' script. After Peak Pairs Analysis (PPA) in digital micrograph program, W atoms (cyan), 2Se atoms (yellow), V atoms (red), and mono Se-vacancies (blue) are clearly distinguished. The Se vacancies paired with V atoms (VSe) were also revealed.

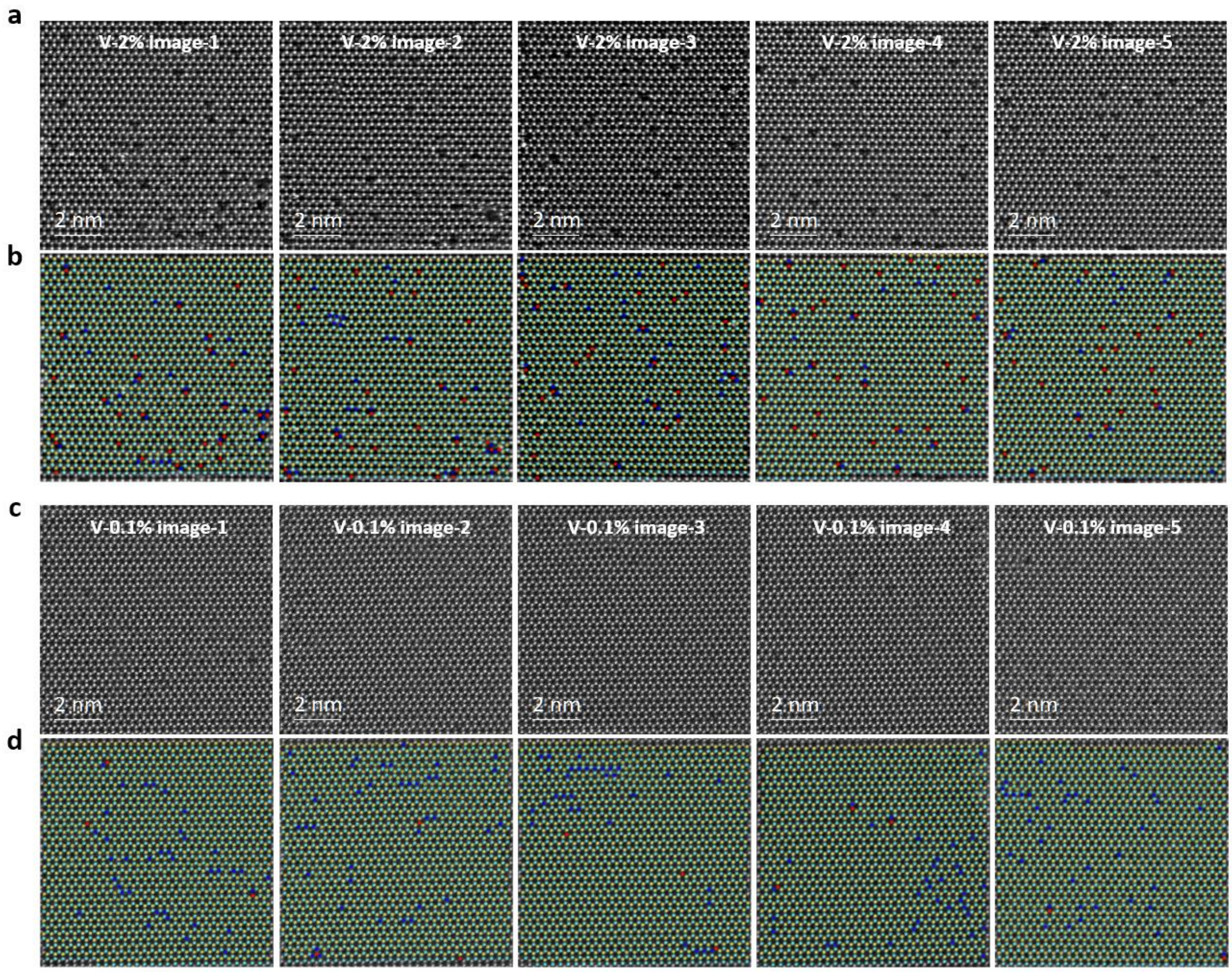

Figure S13. Atom site-wise mapping of 0.1% and 2% V-doped WSe_2 monolayer. Weiner-filtered ADF-STEM images of (a) 2% and (c) 0.1% V-doped WSe_2. False-colored STEM images of (b) 2% and (d) 0.1% V-doped WSe_2.

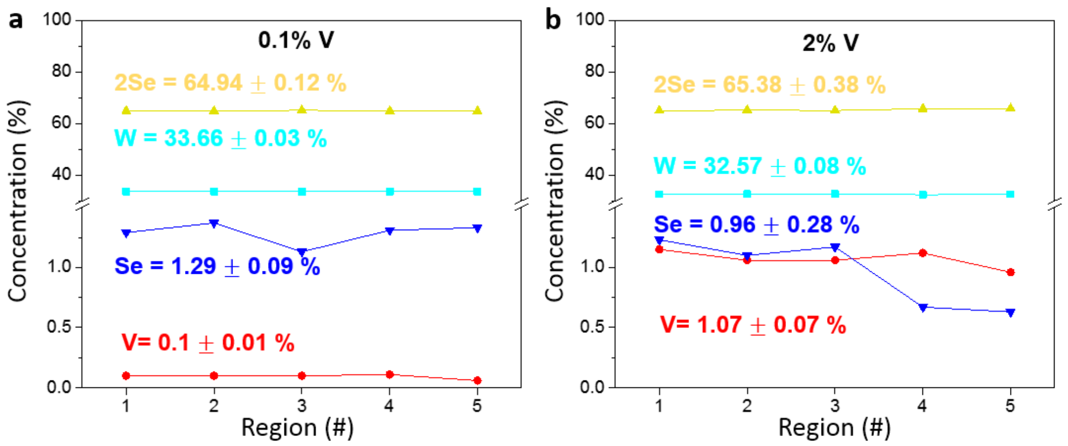

Figure S14. Concentration of W, V, 2Se, and Se (mono Se-vacancy) in V-doped WSe_2. For reliable statistics, we extracted the concentration values from five regions (presented in fig. S11) for each (a) 0.1% and (b) 2% V-doped WSe_2.

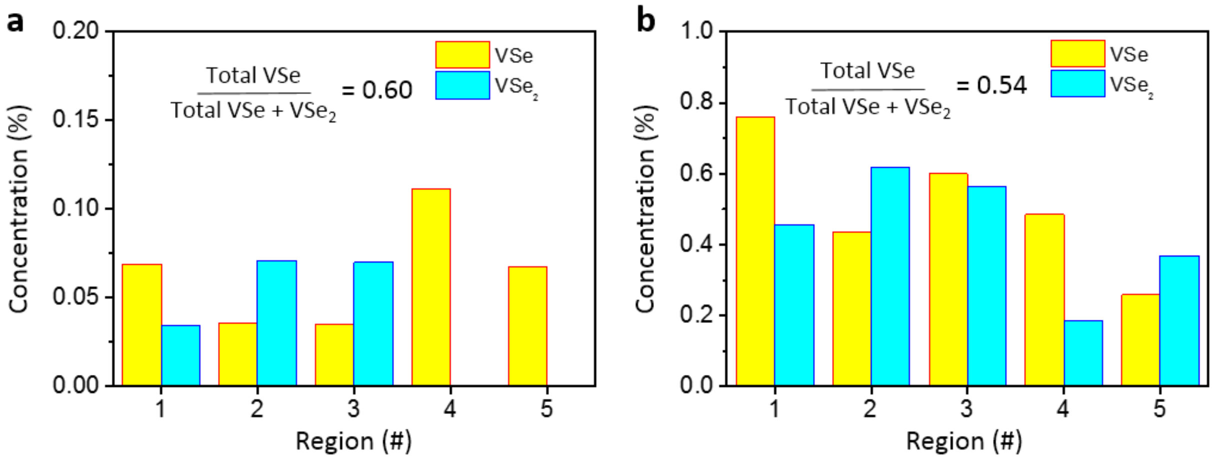

Figure S15. Concentration of VSe and VSe_2 in (a) 0.1% and (b) 2% V-doped WSe_2.

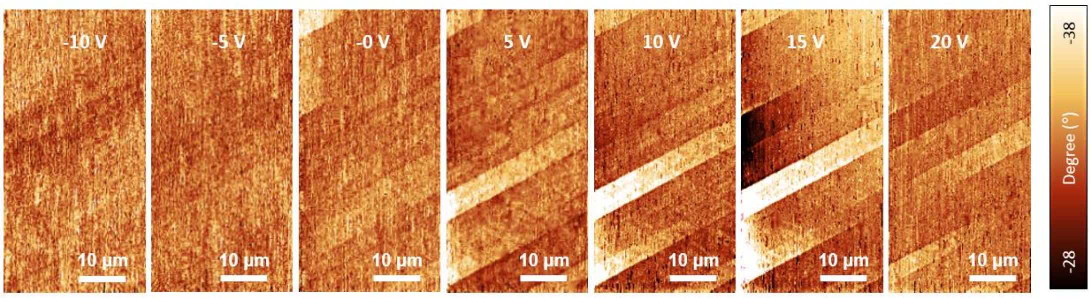

Figure S16. Gate-dependent MFM images for 0.1% V-doped WSe_2 at room temperature.

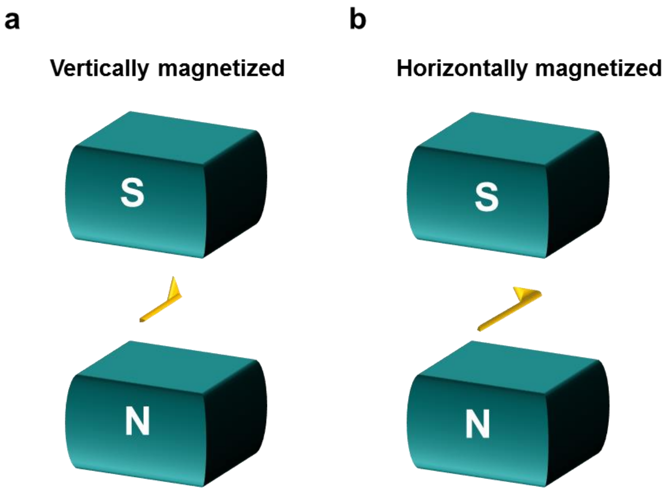

Figure S17. Magnetization of MFM tips with (a) vertical and (b) horizontal directions.

References

[1]. https://www.qdusa.com/sitedocs/appNotes/vsmappnote_5-09.pdf.

[2]. M. Buchner, K. Höflera, B. Henne, V. Ney, and A. Ney, *J. Appl. Phys.* **2018,** 124, 161101.

[3]. K. L. Babcock, V. B. Elings, J. Shi, D. D. Awschalom, and M. Dugas, *Appl. Phys. Lett.* **1966,** 69, 705.
